# Can Deep Learning Using Weight Bearing Knee Anterio-Posterior Radiograph Alone Replace a Whole-Leg Radiograph in the Interpretation of Weight Bearing Line Ratio?

**DOI:** 10.3390/jcm10081772

**Published:** 2021-04-19

**Authors:** Hyun-Doo Moon, Han-Gyeol Choi, Kyong-Joon Lee, Dong-Jun Choi, Hyun-Jin Yoo, Yong-Seuk Lee

**Affiliations:** 1 Department of Orthopedic Surgery, Seoul National University College of Medicine, Seoul National University Bundang Hospital, Seongnam 13590, Korea; hyundoo12@snu.ac.kr (H.-D.M.); meinmed87@naver.com (H.-G.C.); yoo15love@gmail.com (H.-J.Y.); 2 Department of Medicine, Seoul National University College of Medicine, Seoul 03080, Korea; 3 Department of Orthopedic Surgery, Yonsei Sulgee Hospital, Seoul 04707, Korea; 4 Department of Radiology, Seoul National University College of Medicine, Seoul National University Bundang Hospital, Seongnam 13590, Korea; kjoon31@gmail.com (K.-J.L.); chzze4582@gmail.com (D.-J.C.)

**Keywords:** knee, osteoarthritis, deep learning, alignment, weight bearing line

## Abstract

Weight bearing whole-leg radiograph (WLR) is essential to assess lower limb alignment such as weight bearing line (WBL) ratio. The purpose of this study was to develop a deep learning (DL) model that predicts the WBL ratio using knee standing AP alone. Total of 3997 knee AP & WLRs were used. WBL ratio was used for labeling and analysis of prediction accuracy. The WBL ratio was divided into seven categories (0, 0.1, 0.2, 0.3, 0.4, 0.5, and 0.6). After training, performance of the DL model was evaluated. Final performance was evaluated using 386 subjects as a test set. Cumulative score (CS) within error range 0.1 was set with showing maximum CS in the validation set (95% CI, 0.924–0.970). In the test set, mean absolute error was 0.054 (95% CI, 0.048–0.061) and CS was 0.951 (95% CI, 0.924–0.970). Developed DL algorithm could predict the WBL ratio on knee standing AP alone with comparable accuracy as the degree primary physician can assess the alignment. It can be the basis for developing an automated lower limb alignment assessment tool that can be used easily and cost-effectively in primary clinics.

## 1. Introduction

Osteoarthritis (OA), characterized by the degeneration of joints, is the single most common cause of disability in the elderly [1]. The disease burden of knee OA is quite high; OA ranked the eleventh burden of disease measured by the years lost due to disability (YLD) and knee OA accounts for approximately 85% of the burden [2,3]. OA can develop due to wear and tear on any joint, and the joints that bear our weight such as the knee joint are more susceptible [4]. In the knee joint, the medial compartment is the most commonly affected, and accompanying varus malalignment that serves as an important risk factor of development and progression of the disease with vicious cycle [5,6,7]. Since varus malalignment increases the load on the medial compartment, the knee joint with severe medial OA is inevitably more vulnerable to progression of varus malalignment [7,8,9]. Therefore, for diagnosing and follow-up of the knee OA patients, assessment of the alignment is essential.

To assess lower limb alignment, several radiologic parameters are commonly used, and all of which are measured using weight bearing whole-leg radiograph (WLR) such as hip-knee-ankle angle, mechanical axis, and weight bearing line (WBL) ratio [10,11,12]. Among those parameters, the WBL ratio is commonly used for the assessment of the direction of the biomechanical force exerted through the lower limbs in the standing position [9,12]. In addition, the WBL ratio can be used for preoperative planning and postoperative follow-up assessment in alignment correction surgery [10,11,13,14]. However, measuring the WBL ratio is cumbersome in primary clinics because specialized equipment and cost-demanding software are needed to obtain weight bearing WLR [15,16]. Therefore, there have been several studies on whether information about alignment can be obtained with a simple standing knee radiograph only [9,17].

Machine learning, a kind of artificial intelligence method that can learn complex relationships or patterns from empirical data, can produce mathematical models linking several covariates (e.g., radiologic image data in pixels) to some target variable of interest (e.g., radiologic parameters) [18,19,20]. Recently, the convolutional neural network (CNN), one of the deep learning (DL) algorithms, a subset of machine learning, has shown ground-breaking results in image recognition and classification in the medical field [18,19,20,21]. Among orthopedic studies related to the knee joint, many of them using CNN were for predicting the OA stage, however, to the best of our knowledge, there has been little study for the prediction of the alignment [21,22].

It would be generally used in primary clinics easily and cost-effectively if there is a DL model that can automatically assess the alignment of the lower limb with a simple knee standing AP alone to some extent. Therefore, this study was performed to assess lower limb alignment by predicting the WBL ratio using a simple standing knee anterposterior (AP) radiograph alone by adding DL. The purpose of this study was to develop a DL model that predicts the WBL ratio using knee standing AP alone. The hypothesis of this study was that developed DL model would be able to predict the WBL ratio using knee standing AP radiograph alone up to degree that primary physicians can use in the alignment assessment without WLRs.

## 2. Materials and Methods

### 2.1. Dataset

From March 2003 to May 2018, a total of 72,258 patients suffering from knee pain and who subsequently underwent standing knee AP radiographs were searched by the clinical data warehouse in our hospital. Among them, 2763 patients (4% of standing knee AP acquisition) who acquired weight bearing WLR were included. The exclusion criteria are as follows: (1) children with remaining growth plates; (2) previous joint replacement surgery (hip or knee or ankle joint); (3) previous alignment correction surgery; (4) patients with deformity due to previous trauma or congenital diseases. After excluding patients that meet the exclusion criteria, finally 2001 patients with 3997 knee AP radiographs were randomly selected using stratified random sampling [23]. To avoid cluster effect between multiple radiographs in a single patient, only the initial knee AP radiograph was used.

Images whose radiographs were taken after 2017 were used as test sets in order to split the dataset. Images whose radiographs were taken before 2017 were subjected to stratified random sampling at a ratio of 9:1 in proportion to the distribution of WBL ratio, and used as the training set and validation set, respectively.

### 2.2. WBL Ratio Measurement and Labeling

**Figure 1:**
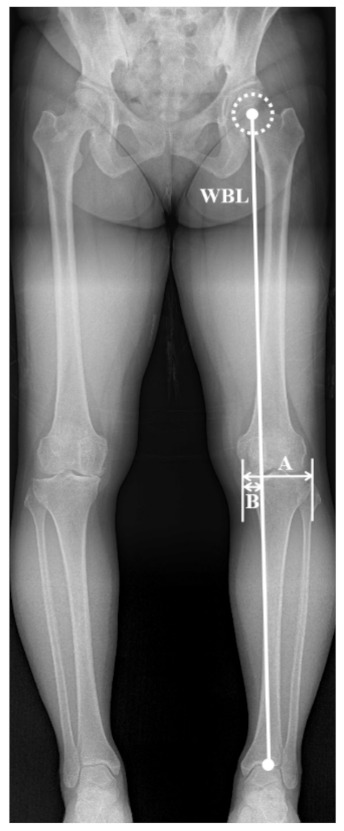
Radiographs showing measurement method of weight bearing line (WBL) ratio. The WBL is detected by drawing a line from the center of the femoral head to the middle point of the proximal talar joint surface. The WBL ratio is calculated as the horizontal distance from the WBL to the medial edge of the tibial plateau (**B**), divided by the width of the tibial plateau (**A**), hence **B**/**A**.

WBL ratio was measured using the weight bearing WLR of all 2001 patients with 3997 knees for labeling of the training set and analysis of prediction accuracy in the validation and test set. The WBL was drawn from the center of the femoral head to the center of the superior articular surface of the talus. The WBL ratio was calculated as the ratio of the crossing point of the mechanical axis, from the medial edge of the tibial plateau to the entire width of the tibial plateau (with the medial tibial edge at 0 and the lateral tibial edge at 1, Figure 1) [24]. The calculated WBL ratio, which is a continuous variable, was rounded to the first decimal place and divided by 0.1 to reconstruct a total of seven categories (0, 0.1, 0.2, 0.3, 0.4, 0.5, and 0.6). Since the number of samples with a WBL ratio less than 0 or more than 0.6 was not large enough, they were included in the 0 and 0.6 categories, respectively. All the measurements were reviewed at 6-week intervals by two blinded independent observers. After then, all anonymized Digital Imaging and Communications in Medicine (DICOM) files of standing knee AP radiograph of the enrolled patients were downloaded from the picture archiving and communication system (PACS) and used. All radiographs were labeled according to the previously categorized WBL ratio.

### 2.3. Image Preprocessing

PyDicom library (version 1.3.0) was used for the preprocessing of DICOM images. The right or left knee was cropped respectively in a knee radiograph that included both knees. The cropped size was 36 × 20 cm^2^, and it was resized to 360 × 200 pixels using bilinear interpolation. The images of the training set were applied with shift augmentation and left and right flipping in order to improve the performance of the algorithm.

### 2.4. DL Algorithm

**Figure 2:**
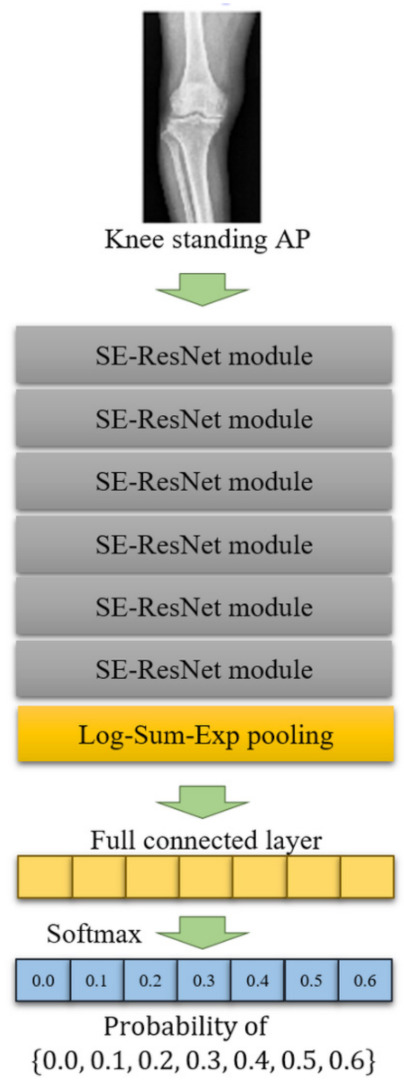
The constructed architecture of the deep learning algorithm. The algorithm was composed by stacking 6 SE-ResNet blocks, and Log-Sum-Exp pooling, full connected layer, and Softmax activation functions were sequentially applied the last Se-ResNet block.

We developed a deep learning algorithm based on the TensorFlow library (version 1.12) on a Linux operating system (Ubuntu 16.04) with CUDA/cuDNN (version 9.0 and 7.6, respectively) installed. The graphic processing unit used to train the algorithm was an Nvidia Geforce GTX Titan Xp. The CNN we designed was constructed based on the residual block to which squeeze-and-excitation attention (SE-ResNet block) was applied [25,26]. CNN was composed by stacking six SE-ResNet blocks, and Log-Sum-Exp pooling, full connected layer, and Softmax activation functions were sequentially applied to the last Se-ResNet block (Figure 2) [27]. As a result of calculating Softmax function, the probability of WBL ratio (0, 0.1, 0.2, 0.3, 0.4, 0.5, and 0.6) was calculated. The output of the CNN we developed was a discrete probability distribution corresponding to seven WBL ratio intervals, and the WBL ratio of the test image was predicted as the mean of this distribution [28]. WBL ratio was predicted by rounding the mean of distribution to one decimal place. Xavier initialization was applied as the initial weight of CNN [29]. During the training procedure, the learning rate, decay rate, and decay steps were set to 0.001, 0.94, and 5000, respectively. The mini-batch size was set to 4. Cross-entropy was considered as the objective function, and mean loss and variance loss were added along with cross-entropy to correct ambiguity of the WBL ratio [28]. RMSProp optimizer was used as the optimizer to minimize the objective function [30]. To prevent overfitting of the CNN, L2 regularization was applied to the weights.

Gradient-weighted class activation mapping (Grad-CAM) was used to find out which part of the CNN predicted the WBL ratio [31]. Grad-CAM provides a coarse localization map highlighting important regions in the image for predicting the concept of the developed CNN models [31]. Grad-CAM was calculated as a linear combination between the gradient of the layer immediately before Log-Sum-Exp pooling and the corresponding layer. Rectified linear unit (ReLU) activation was applied to the linear combination to highlight the positive effects on each WBL ratio. Grad-CAM corresponding to seven WBL ratio intervals was calculated respectively, and it was confirmed which region was sensitive to the input image.

### 2.5. Statistical Analysis

All statistical analyses were performed using R statistical software, version 3.6.3 (The R Foundation for Statistical Computing, Vienna, Austria). The data were presented as means and standard deviations for continuous variables. One-way analysis of variance was performed to compare the quantitative variables (i.e., age, body mass index (BMI), and WBL ratio). Pearson chi-squared test or Fisher’s exact test were used to comparing the qualitative variables (i.e., gender). *p*-value < 0.05 were considered statistically significant.

Mean absolute error (MAE) and cumulative score (CS) was used as a measure to find out how well our CNN fits the WBL ratio [28]. MAE is defined as the mean absolute error between the estimated WBL ratio and ground-truth WBL ratio, $MAE=\frac{1}{N}\sum_{i=1}^{N}\left|\hat{y_{i}}-y_{i}\right|$, with $\hat{y_{i}}$: estimated WBL ratio of ith data, $y_{i}$: ground-truth WBL ratio of ith data. CS measures the WBL ratio estimation accuracy given a tolerance of absolute error, $CS\left(j\right)=\frac{N_{e\leq j}}{N}\times 100\%$, $N_{e\leq j}$: number of data on which WBL grade estimation makes an absolute error no higher than j. *MAE* is a measure to indicate the difference between the actual measured WBL ratio and the WBL ratio predicted by CNN. The error range *j* should be determined when calculating CS. CS means the ratio of the data whose WBL ratio predicted by CNN among the entire dataset is within the error range *j* with respect to the actually measured WBL ratio. If *j* is 0.0, CS has the same meaning as accuracy. When *j* is 0.1 and 0.0, CS was denoted as CS (0.1) and CS (0.0) and measured for predicted WBL ratio.

## 3. Results

The final inclusion of baseline characteristics of patients and distribution of labels in training, validation, and test sets is summarized in Table 1. Age, gender, and BMI were not significantly different between each dataset. However, the WBL ratio of the test dataset showed significantly higher than other datasets (*p* < 0.001). We selected the weight at which CS (0.1) was the maximum in the validation set during training process and then applied it to the test set. The final model evaluation was performed on 386 subjects.

**Figure 3:**
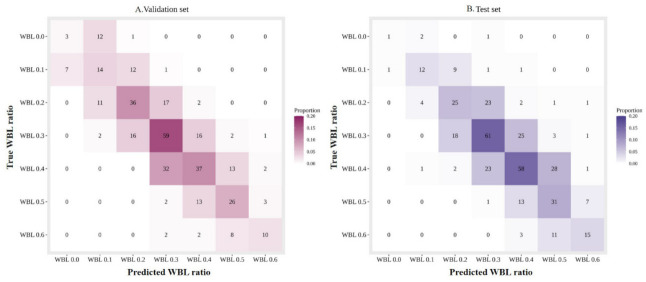
Confusion matrices of predicted and ground truth labels of the WBL ratio. (**A**): validation set, (**B**): test set. WBL, weight bearing line.

In the validation image set, MAE was 0.054 (95% CI, 0.048–0.061) and CS was 0.953 (95% CI, 0.924–0.970) within error range 0.1 and the CS was 0.511 (95% CI, 0.458–0.564) within error range 0.0. In the test set of image data, MAE was 0.054 (95% CI, 0.048–0.061) and CS was 0.951 (95% CI, 0.924–0.970) within error range 0.1 and the CS was 0.526 (95% CI, 0.474–0.577) within error range 0.0 (Table 2). Figure 3 displays the confusion matrices of classification results in the validation and test set. Among the seven categories, the WBL ratio 0.0 has the lowest accuracy with lots of samples predicted to WBL ratio 0.1. The accuracy of the rest categories was much higher.

### Gradient-Weighted Class Activation Mapping (Grad-CAM)

**Figure 4:**
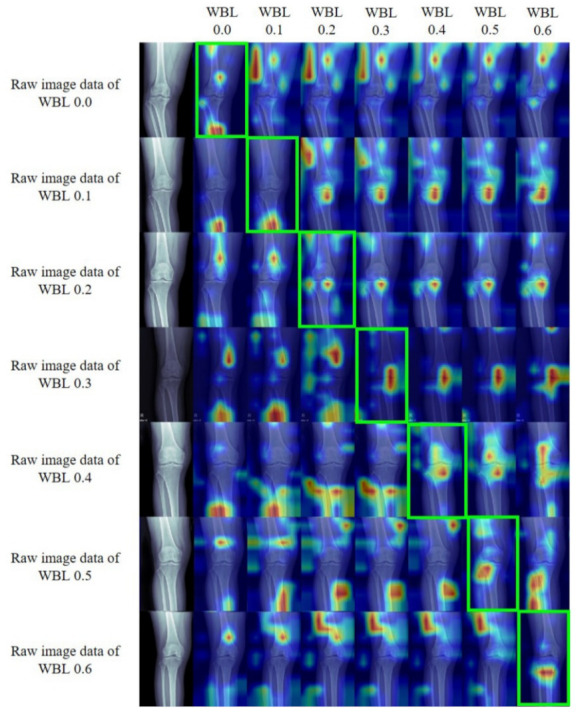
Knee radiographs of each WBL ratio categories which overlaid with the heat maps obtained by using the Grad-CAM method. WBL, weight bearing line; CAM, class activation mapping.

We applied the Grad-CAM technique to locate the most significant areas in the image for classification (Figure 4). They are image data representing a WBL ratio of 0.0 to 0.6. For raw image data of WBL 0.2 to 0.6, the heat map signals appear around the knee joint area. However, in the raw image data of WBL ratio 0.0, the heat map signal appeared on multiple points including femoral diaphysis and metaphysis, fibular head, and tibial diaphysis. In the raw image data of the WBL ratio of 0.1, the heat map signal appeared on the tibial diaphysis. Thus, it would be complicated for the DL algorithm to detect and diagnose the WBL ratio of 0.0 and 0.1 that reveal severe varus malalignment.

## 4. Discussion

The principal findings of this study are as follows. The DL algorithm could interpret the categorical analysis of the WBL ratio of the lower limb from knee standing AP alone even it could not predict the accurate WBL ratio value. The algorithm showed an accuracy of 95.1% as CS, MAE of 0.054. To the best of our knowledge, this is the first study for the interpretation of knee joint alignment using deep learning. This would be meaningful because the developed CNN model could be used as a supportive tool for the primary physician for assessing lower limb alignment in patients with knee OA without special equipment or software that can be only available in the advanced hospital.

The gold standard for assessing lower limb alignment is weight bearing WLR, which can obtain visual information of hip, knee, and ankle joints [9,17]. Since the equipment and software for WLR is far expensive than that for simple radiographs, it is not well prepared in primary clinics [15,16]. Even in the advanced hospital, WLR was only taken 4% of patients who have taken standing knee AP radiograph in our study. Therefore, in many clinics that do not have such equipment and software, the alignment can only be predicted indirectly using a simple knee radiograph [9,32]. However, Lee et al. [9] reported that the severity of varus malalignment could be underestimated in the simple knee AP without WLR and they concluded that it can’t replace WLR. Therefore, it was recommended that when knee OA patients are needed to assess one’s alignment, they should visit an advanced hospital where equips weight bearing WLR.

Several studies tried to enhance the performance and accuracy of making a diagnosis of knee OA using DL algorithms [21,33]. However, most of the previous studies were focused on the classification of knee joint OA according to the Kellgren-Lawrence grade compared with the interpretation of radiologists. In addition, to the best of our knowledge, most of those studies used image data with cropping around the knee joint. In this study, we used the knee standing AP image without cropping around the knee joint due to make the validation possible for use in the primary clinics. If the developed CNN model could alternate WLR in assessing lower limb alignment, it would be helpful for knee OA who is not well-approachable to metropolitan areas, especially for those who have a physical disability for the disease.

The strength of this study is that it is the first study to use DL to predict lower limb alignment. This research result will be the basis for the development of a cost-effective and easily available alignment prediction DL model that can be used in primary clinics in the future. This study has also several limitations. First, number of patients who have WLR was too small compared to the number of patients who have simple knee AP radiograph. However, as mentioned, WLR is not routinely acquired in patients with knee pain because the equipment is expensive and difficult to access. This is more prominent in the primary clinic. Second, it was difficult to interpret the developed CNN model because the Grad-CAM highlighted inconsistent areas or areas outside the body, especially in the subjects with severe varus deformity (WBL ratio 0.0 and 0.1). This is thought to be due to insufficient number of subjects with relatively severe varus deformity. Therefore, it was assumed that new methodological approaches that combines CNN with ML such as deformity profiles would make Grad-CAM highlights consistent areas. Third, the number of subjects was not enough to build the model for accurate prediction. As a result, it was possible to predict the approximate information of the WBL ratio only within the seven categories. These results may be useful for predicting the approximate location of the WBL ratio of patients visiting primary clinics, but it would be difficult to use for preoperative planning and postoperative follow-up, which require precise numbers. If a sufficient number of subjects is secured and the algorithm is reinforced in a future study, it can be possible to use for preoperative planning of alignment correction surgery. Fourth, the number of test sets was small although the ratio of validation and test set was adequate.

## 5. Conclusions

Developed DL algorithm could predict the WBL ratio on simple knee standing AP alone with comparable accuracy as the degree primary physician can assess the alignment. It can be the basis for developing an automated lower limb alignment assessment tool that can be used easily and cost-effectively in primary clinics.

## Figures and Tables

**Table 1:** Baseline characteristics.

	Training Set	Validation Set	Test Set	Total	*p*-Value
Age (year)	64.8 ± 9.22	64.3 ± 9.41	65.1 ± 9.02	64.8 ± 12.8	0.449
Gender (M/F)	573/2676	61/301	82/304	716/3281	0.186
BMI (kg/m^2^)	25.6 ± 3.17	25.7 ± 2.49	25.4 ± 2.42	25.6 ± 3.10	0.284
WBL ratio	0.32 ± 0.16	0.32 ± 0.17	0.35 ± 0.14	0.32 ± 0.16	<0.001 *
0.0	148	16	4	168	
0.1	307	34	24	365	
0.2	595	66	56	717	
0.3	859	96	108	1063	
0.4	754	84	113	951	
0.5	392	44	52	488	
0.6	194	22	29	245	
Total	3249	362	386	3997	

Values are presented as number or mean ± standard deviation. WBL, weight bearing line; *, statistically significant.

**Table 2:** Results for prediction of WBL ratio.

	Validation Set	Test Set
MAE	0.054	0.054
CS (0.1)	0.953 (345/362, 0.924–0.970)	0.951 (367/386, 0.924–0.970)
CS (0.0)	0.511 (185/362, 0.458–0.564)	0.526 (203/386, 0.474–0.577)

MAE: Mean Absolute Error, CS: Cumulative Score, CS (0.1) and CS (0.0) are proportions, nominator/denominator and 95% confidence interval in the parentheses.
